# Development and effectiveness evaluation of a mobile health-based follow-up management model for patients after hematopoietic stem cell transplantation

**DOI:** 10.3389/fmed.2026.1786747

**Published:** 2026-03-19

**Authors:** Xiaoyi Zhu, Luling Zhou, Siyu Zong, Hongyan Li, Shuang Guo, Lulu Zhu, Dongliu Li

**Affiliations:** 1 Department of Hematology, Chengdu Boe Hospital, Chengdu, China; 2 Nursing Department, Chengdu Boe Hospital, Chengdu, China; 3 School of Medicine, Yan'an University, Yan'an, China; 4 Department of Oncology, The Tenth Affiliated Hospital, Southern Medical University (Dongguan People’s Hospital), Dongguan, Guangdong, China

**Keywords:** complication prediction, hematopoietic stem cell transplantation, mobile health, quality of life, self-management

## Abstract

**Objective:**

To evaluate the association between mobile health (mHealth)–based follow-up and quality of life, self-management capacity, and complication control in patients after hematopoietic stem cell transplantation (HSCT).

**Methods:**

This retrospective single-center cohort study included 310 HSCT recipients (January 2020–December 2024). Patients were assigned to an mHealth intervention group (*n* = 145) or standard-care group (*n* = 165). After 1:1 propensity score matching (caliper = 0.2), 248 balanced patients (124 per group) were analyzed. The control group received routine outpatient follow-up and standard education. In addition to routine care, the intervention group received 6-month mHealth-based management program incorporating real-time health monitoring with automated alerts, personalized education, medication management, and online professional support. Patients could upload health data in real time and consult medical staff online through the APP. Primary outcomes included quality of life, self-efficacy, and complication incidence; secondary outcomes included symptom management, satisfaction, laboratory parameters, and healthcare utilization. Multivariable regression and subgroup analyses were conducted to assess independent associations and consistency of findings.

**Results:**

At 6 months, the mHealth group showed significantly greater improvements in quality of life (EORTC +10.0 points, *p* < 0.001) and self-efficacy (+2.01 points, *p* < 0.001). The incidence of infection, cytomegalovirus (CMV) reactivation, and emotional disorders was lower in the intervention group (all *p* < 0.05). Among allogeneic recipients, mHealth management was associated with reduced graft-versus-host disease (GVHD) and CMV reactivation. Secondary outcomes were associated with better symptom management, higher patient satisfaction, favorable changes in immune and renal function indicators, and lower readmission rates and nursing visits (all *p* < 0.01). CD4 count and symptom management scores were independently associated with better quality of life and self-efficacy, whereas chronic comorbidities were associated with poorer outcomes. Higher BMI, CD4 level, and monitoring capacity were associated with a lower risk of complications. These observed associations remained consistent across subgroups. Engagement analysis indicated predominant use of monitoring functions; low participation (14.5%) was more common among older, less educated, and allogeneic transplant patients.

**Conclusion:**

mHealth-based remote follow-up was associated with favorable changes in quality of life, self-efficacy, and complication rates after HSCT. While findings suggest potential clinical feasibility and scalability, causal inference is markedly limited by the retrospective design and inherent selection bias.

## 1 Introduction

Hematopoietic stem cell transplantation (HSCT) is the optimal and effective treatment for various hematologic malignancies, severe aplastic anemia, and immune system disorders (1). More than one million HSCT procedures have been performed globally (2), and nearly 60,000 HSCTs have been completed in China over the past decade (3). Over 70% of HSCT patients are expected to survive long-term (4). However, the clinical management of HSCT patients is characterized by significant heterogeneity, with notable differences in clinical burden, psychological impact, and self-management requirements between allogeneic and autologous transplant recipients: 70 to 90% of allogeneic HSCT patients need to cope with long-term complications such as graft-versus-host disease (GVHD) and severe infections (5), requiring lifelong rigorous follow-up. The focus of self-management for these patients lies in GVHD monitoring, standardized use of immunosuppressants, and infection prevention (6–8). In contrast, autologous transplant patients have no risk of GVHD, with complications mainly including infections and liver or renal function impairment; their follow-up period is shorter, and the core of self-management is postoperative rehabilitation, medication adherence, and health monitoring (8, 9).

Due to the complexity of treatment regimens and long recovery period (6 to 12 months), medication and dietary adherence are particularly important for HSCT patients (10). Studies on HSCT adherence have reported a significant decline in patients’ adherence to various oral medications within months after discharge, and this issue has been linked to medication complexity and increased follow-up requirements (11, 12). Compared with patients with common chronic diseases, HSCT patients face polypharmacy, higher follow-up frequency, and more arduous self-management tasks, making it difficult for conventional intervention models to directly meet their specific needs (13). However, existing intervention measures (such as traditional outpatient follow-up and verbal health education) have not effectively addressed the problem of poor adherence, and numerous challenges remain in improving self-management ability and reducing complications: single routine education is generally insufficient to enhance long-term adherence, highlighting the need for dynamic monitoring and interdisciplinary comprehensive intervention for HSCT patients (14, 15). Such interventions are associated with long follow-up intervals and delayed feedback, failing to meet the dynamic monitoring needs of HSCT patients and resulting in less favorable outcomes compared with the general population (13).

Mobile health (mHealth) refers to the use of mobile communication technologies, such as smartphones and personal digital assistants (PDAs), to provide public health services, healthcare management, and to track personal patient information (16). In recent years, the development of mHealth technologies has led to the emergence of health management platforms based on smartphones and wearable devices. These platforms allow for real-time monitoring of patient health, reminders to adhere to treatment protocols, and personalized advice, which are expected to improve patient adherence and health outcomes (17, 18).

In recent years, there has indeed been an increase in digital intervention studies targeting HSCT recipients, but these studies have several notable limitations: most existing studies focus on a single functional module (e.g., exercise/activity tracking, symptom reporting, or psychological/caregiver support alone), lacking integrated platforms that cover multiple dimensions such as dynamic complication monitoring, medication management, and rehabilitation (19, 20). Some studies/trials only include recipients of a single transplant type or primarily target caregivers (e.g., evaluating only an autologous/allogeneic subgroup or using caregivers as the sole sample), thereby limiting the generalizability of conclusions to the entire HSCT population (21). In addition, the majority of existing interventions are early small-sample or pilot studies with generally short follow-up periods (most outcome evaluations focus on the short term after intervention, ≤ 3 months), and long-term intervention effects and sustained outcome improvements have not been systematically assessed (22, 23). Compared with digital health interventions for other clinical populations, HSCT patients require simultaneous monitoring of complications (such as acute/chronic GVHD), assessment of immune function status, and guidance on individualized immunosuppressive/anti-infective strategies, which places higher demands on the professionalism, real-time performance, and clinical operability of the platform. Existing studies/products are insufficient in these aspects, emphasizing the need to develop platforms with real-time monitoring, clinical decision support, and interdisciplinary connection capabilities (22, 24).

However, current research primarily focuses on specific diseases or single-dimensional intervention effects, with a lack of systematic evaluations of comprehensive management platforms in multi-dimensional interventions (25, 26), especially in the context of HSCT patients. Although studies have shown that mHealth platforms can effectively improve medication adherence and health behaviors, their comprehensive impact on quality of life, complication incidence, and patient satisfaction has not been fully explored (27, 28). Most existing studies are limited to short-term outcomes and do not consider the potential impact of personalized platform features and patient interaction models on treatment outcomes (29). Furthermore, HSCT-related studies generally lack subgroup stratified analysis between autologous and allogeneic transplantation, making it difficult to accurately match the self-management needs of patients with different transplant types (most eHealth/mHealth studies on hematopoietic stem cell transplantation (HCT/HSCT) are small-sample pilot/feasibility studies, and the intervention objects or samples often target a single transplant type or early post-discharge follow-up population, without sufficient stratified comparison and long-term evaluation of the two types of transplant recipients) (19, 22).

Therefore, this study aims to explore the impact of mobile health management platform intervention on self-management ability, quality of life, complication incidence, and satisfaction of HSCT patients, while taking into account the management differences between the two transplant types to improve the clinical adaptability of the study results. We hypothesize that personalized mHealth platforms will significantly improve self-management behaviors, quality of life, and reduce the occurrence of relevant complications. Additionally, this study will compare clinical and quality of life indicators between the intervention and control groups to provide scientific evidence for the widespread clinical application of mHealth platforms.

## 2 Materials and methods

### 2.1 Study population

This was a retrospective, single-center cohort study based on real-world data from patients who underwent hematopoietic stem cell transplantation (HSCT) at a tertiary hospital between January 1, 2020, and December 31, 2024. The study aimed to evaluate the impact of mobile health (mHealth)-based follow-up management in the post-HSCT population. Data were extracted from electronic medical records, the nursing information system, and the institutional mHealth platform.

The study was approved by the hospital’s ethics committee [Approval No.: (2024060)] and conducted in accordance with the Declaration of Helsinki and Good Clinical Practice (GCP) guidelines. As a retrospective analysis, informed consent was waived by the ethics board.

The inclusion criteria for this study were as follows: age ≥ 18 years, regardless of gender; having received autologous or allogeneic hematopoietic stem cell transplantation (HSCT) between January 2020 and December 2024, and entering a stable phase at 3 months after transplantation (defined as 90 full days from the date of stem cell infusion); completing at least 6 months of standardized follow-up (the follow-up period started on the day of 3 months after transplantation and ended on the day of 9 months after transplantation) with complete follow-up records; having the ability to use a smartphone and mobile application (APP) and possessing basic self-management skills; having complete usage records on the hospital-based mobile health (mHealth) platform or clearly not having used the platform.

The study time points and evaluation protocol were clearly defined to ensure complete comparability between the two groups: ① Baseline evaluation: conducted on the day of 3 months after transplantation for all patients, including assessments of demographic characteristics, clinical indicators, quality of life, self-efficacy, self-management ability, and adherence-related indicators; ② Endpoint evaluation: conducted on the day of 9 months after transplantation; Intervention period: the mHealth group initiated the intervention on the day of baseline evaluation and continued until the day of endpoint evaluation, with a total duration of 6 months; the control group received routine follow-up during the same period, and the evaluation time, content, and procedures were completely consistent between the two groups.

The exclusion criteria were: occurrence of acute graft-versus-host disease (GVHD) (≥ grade II), severe infection, or readmission due to other complications within 6 months after transplantation; presence of mental illness, severe cognitive impairment, or other conditions that did not meet the requirements for long-term follow-up; pregnancy or lactation period; severe missing sample data or failure to complete the full 6-month follow-up from 3 months to 9 months after transplantation.

A total of 368 HSCT patients were screened in this study, among which 58 patients were excluded due to failure to meet the inclusion/exclusion criteria (accounting for 15.8% of the total screened population), and 310 patients were finally included for preliminary analysis. After 1:1 propensity score matching (PSM), a total of 248 patients (124 in the mHealth intervention group and 124 in the control group) were included in the statistical analysis. Among the 58 excluded patients, 8 were excluded due to lack of basic technical skills such as smartphone operation and APP usage, accounting for 13.8% of the total excluded population and 2.2% of the total screened population.

### 2.2 Grouping and intervention protocol

A multidisciplinary team was established in this study to participate in project design, intervention implementation, and outcome evaluation. The team consisted of 3 attending physicians specializing in HSCT, 4 specialist nurses, 2 clinical pharmacists, 1 rehabilitation therapist, and 1 psychological therapist, covering fields such as clinical diagnosis and treatment, nursing care, medication guidance, rehabilitation intervention, and psychological support. The time invested by clinical staff in the project was quantified and standardized: attending physicians fixed 4 h per month for data review, remote consultation, and protocol adjustment, while specialist nurses fixed 8 h per month for health guidance, follow-up management, and health education delivery. All the above-mentioned time was incorporated into their regular working hours. Personnel with different professional backgrounds participated in accordance with their job responsibilities: specialist nurses undertook the main work of daily follow-up, attending physicians were responsible for clinical decision-making and risk intervention, and pharmacists, rehabilitation therapists, and psychological therapists provided specialized support, with their participation level matching their job responsibilities.

During the design, preliminary development, and platform tool selection and integration stages of the mHealth application, the actual needs of medical professionals, patients, and caregivers were fully incorporated through questionnaires and focus group interviews: suggestions from 15 HSCT specialist medical staff on monitoring dimensions, early warning processes, and operational convenience were collected, as well as needs from 30 patients and 20 caregivers on interface simplicity, readability of health education materials, and care coordination functions. These needs were integrated into the entire process of program development and platform construction to ensure that the tool design was suitable for both clinical and home care scenarios.

Patients were divided into the intervention group (mHealth group) and the control group (routine group) based on whether they received mHealth follow-up intervention. Patients in the mHealth group registered and used the mHealth platform on the day of baseline evaluation (3 months after transplantation) and continued to use it until the end of follow-up (on the day of 9 months after transplantation, i.e., after 6 months of intervention/follow-up). Patients in the control group did not use the mHealth platform during the same period and only received traditional offline outpatient follow-up, with the same follow-up cycle and evaluation time points as the intervention group. During the monthly outpatient follow-up, physicians evaluated patients’ clinical indicators, symptoms, and treatment effects, and provided routine health education, medication guidance, and lifestyle suggestions.

A total of 310 eligible HSCT patients were included in the study, including 145 in the intervention group and 165 in the control group. To reduce bias caused by baseline differences, 1:1 propensity score matching (PSM) was used for comparative analysis.

#### 2.2.1 Health data monitoring and feedback

In the intervention group, patients were provided with personalized mHealth devices, such as smart blood pressure monitors, thermometers, glucometers, and weighing scales. Health parameters were recorded and uploaded via the app in real-time. Blood pressure and heart rate were monitored four times daily (morning, pre-lunch, pre-dinner, and bedtime). Alerts were automatically triggered for abnormal readings, prompting clinician and patient notifications. Body temperature and weight were tracked daily to detect early signs of infection or GVHD.

The platform synthesized data from health records and generated trend reports to identify deterioration risks and recommend clinical visits. Blood glucose data were integrated with diet, physical activity, and medication logs to provide individualized feedback and adjustment suggestions.

All health-related data collected from patients in the intervention group were uploaded in real time to a centralized hospital data platform. Clinicians had continuous access to patients’ physiological parameters and were able to intervene promptly as needed. The platform not only aggregated longitudinal health data but also employed machine learning algorithms to analyze trends and forecast potential health risks. When any parameter exceeded predefined thresholds, the system automatically generated alerts, notifying both the patient and the relevant clinical team.

Upon receiving alerts, physicians could contact the patient directly, assess clinical status, and provide timely medical advice. Additionally, personalized monthly health reports were generated for each patient and made accessible via the mHealth app. These reports displayed trends in key indicators such as blood pressure, body temperature, and weight, and offered personalized lifestyle recommendations accordingly. Each report also included physician-generated health advice, medication adjustments, and tailored exercise plans.

#### 2.2.2 Personalized health education and interventions

Based on patients’ individual health profiles, clinical history, and post-transplant recovery trajectories, the mHealth platform automatically delivered personalized health education modules. These modules included:

Prevention and early recognition of transplant-related complications, such as graft-versus-host disease (GVHD) and adverse effects of immunosuppressive therapy.
Nutritional guidance tailored to real-time data on blood glucose, blood pressure, and body weight.
Exercise recommendations adjusted according to physiological status, as recorded by smart devices including glucometers and digital scales.
Mental health support, addressing common post-transplant challenges such as anxiety and depression, with regular push notifications providing cognitive-behavioral strategies and emotional self-regulation techniques.
Patients were able to consult healthcare providers through the in-app messaging feature. Whenever symptoms or concerns arose, they could directly contact their care team for real-time guidance, ensuring continuity of care beyond in-person visits.

#### 2.2.3 Intelligent medication management and optimization

Given the critical role of pharmacotherapy in HSCT recovery, the mHealth platform incorporated intelligent medication management functions. Patients recorded daily medication intake through the app, which automatically tracked adherence and generated reports on medication compliance. In cases of missed or delayed doses, the system issued reminders and provided guidance for dose adjustments.

Clinicians were able to monitor adherence patterns and physiological responses in real time, allowing for data-driven medication titration. This functionality enabled proactive management of immunosuppressive regimens and reduced the risk of drug-related complications.

#### 2.2.4 Psychosocial support and peer networking

The mHealth application featured a virtual community space where patients could join specific peer groups—such as those undergoing autologous or allogeneic transplantation—to share experiences, offer mutual support, and exchange coping strategies. The platform also regularly delivered psychological counseling modules, helping patients address emotional issues commonly encountered during recovery, including anxiety and depression.

For patients requiring additional support, the platform offered the option to schedule one-on-one virtual counseling sessions with physicians or mental health professionals, thereby integrating psychosocial care into routine disease management.

### 2.3 Data collection and outcome measures

The measurement time points and collection methods of all evaluation indicators were identical between the two groups to ensure inter-group comparability and the internal validity of the study. The evaluation time points were uniformly set as follows: ① Baseline evaluation: conducted on the day of 3 months after transplantation (i.e., before the initiation of intervention); ② Endpoint evaluation: conducted on the day of 9 months after transplantation (i.e., after the completion of 6-month intervention/follow-up). All patients in both groups completed the evaluation of all indicators at the above two fixed time points, with completely consistent evaluation procedures, duration, and requirements.

The primary outcome indicators included quality of life, treatment adherence, and complication incidence. Quality of life was assessed using the European Organization for Research and Treatment of Cancer Quality of Life Questionnaire-Core 30 (EORTC QLQ-C30) once at baseline and once at endpoint. It was collected via patient self-report, completed online through the hospital’s nursing information system or mobile health (mHealth) platform. The scale covers multiple dimensions, including physical function, emotional function, social function, fatigue, and pain, with scores ranging from 0 to 100; higher scores indicate better quality of life. Treatment adherence was evaluated using the Self-Efficacy Measure (SME) once at baseline and once at endpoint, also via patient self-report online. The assessment included compliance behaviors such as medication taking, dietary control, exercise, and blood glucose monitoring, with a total score of 12; higher scores indicate better adherence. The incidence of complications was determined by reviewing records in the electronic medical record system, with two specialist medical staff independently counting the occurrences of acute/chronic graft-versus-host disease (GVHD), infections, cytomegalovirus (CMV) reactivation, and other complications during the follow-up period.

The secondary outcome indicators included self-management ability, clinical parameters, and patient satisfaction. Self-management ability was assessed using the Self-Management Questionnaire (SMQ) once at baseline and once at endpoint, via patient self-report online, covering dimensions such as health monitoring, medication use, symptom recording, and emotional management. Clinical laboratory parameters, including white blood cell (WBC) count, hemoglobin (Hb), creatinine (Cr), and CD4 + T lymphocyte count, were collected uniformly at baseline and endpoint. The data were obtained from the hospital’s electronic medical record system, standardized detected and automatically entered by the clinical laboratory, without the need for patients to fill in the information themselves. Patient satisfaction was evaluated using the Patient Satisfaction Questionnaire (PSQ) only at the endpoint, via patient self-report online, to assess the acceptance, user experience, and overall satisfaction with the follow-up management model.

All scale data were completed by patients through independent self-report without any intervention or guidance from medical staff during the entire process. Laboratory data were routine clinical test results, which were objective and standardized. The data collection channels (online system/electronic medical record), filling methods, and evaluators were completely consistent between the two groups, so as to minimize measurement bias.

### 2.4 Statistical analysis

All data were analyzed using SPSS 26.0. Continuous variables were expressed as Mean±SD, with inter-group comparisons by independent samples t-test; categorical variables were presented as frequencies (%), compared using Chi-square test. Statistical significance was set at *p* < 0.05.

To reduce confounding bias and improve inter-group comparability, 1:1 propensity score matching (PSM) was performed between the mHealth and control groups. Matching variables included age, gender, transplant type, primary disease, comorbidities, education level, and BMI. Nearest neighbor matching was adopted with a caliper of 0.2 (without replacement). Balance of baseline variables after matching was evaluated by Standardized Mean Difference (SMD), with SMD < 0.1 indicating successful matching.

Multiple linear regression models were constructed, with EORTC and SME scores at 6-month follow-up as dependent variables, and intervention group (mHealth vs. Control), CD4 endpoint value, comorbidities, BMI, baseline SME/SMQ scores, and education level as independent variables. All continuous variables underwent linear fitting test; skewed data were standardized or logarithmically transformed. Model results were reported as unstandardized regression coefficient (*β*), t-value, and *p*-value; goodness of fit was assessed by R^2^ and residual plots. Collinearity was checked (Variance Inflation Factor, VIF < 5) to ensure variable independence.

Logistic regression was used to evaluate the impact of mHealth intervention on complication risk, with data split into training (70%) and testing (30%) sets. The dependent variable was the occurrence of any complication (infection, CMV reactivation, GVHD, etc.; 0 = none, 1 = yes). Both groups had consistent CMV viremia monitoring (once weekly during stable post-transplant period, increased to 2–3 times weekly for suspected symptoms) to avoid confounding. Potential risk factors were initially screened by univariate regression (*p* < 0.1 included in multivariate analysis), with backward stepwise selection. OR, 95% confidence interval (CI), and *p*-value were reported. Model predictive ability was evaluated by ROC curve (AUC) and Hosmer-Lemeshow test (*p* > 0.05 for good fit); Decision Curve Analysis (DCA) was used to assess net benefit at different decision thresholds.

Prespecified subgroup analyses were conducted to explore intervention applicability, with subgroup variables including age (<45 vs. ≥ 45 years), gender, transplant type (autologous vs. allogeneic), comorbidities, and baseline SME level (median-split). OR and 95%CI of mHealth intervention versus control were calculated for each subgroup. Cochran’s Q test or interaction term analysis (P for interaction) was used to evaluate potential subgroup interactions, with results presented as a forest plot.

## 3 Results

### 3.1 Baseline characteristics before and after propensity score matching

**Figure 1:**
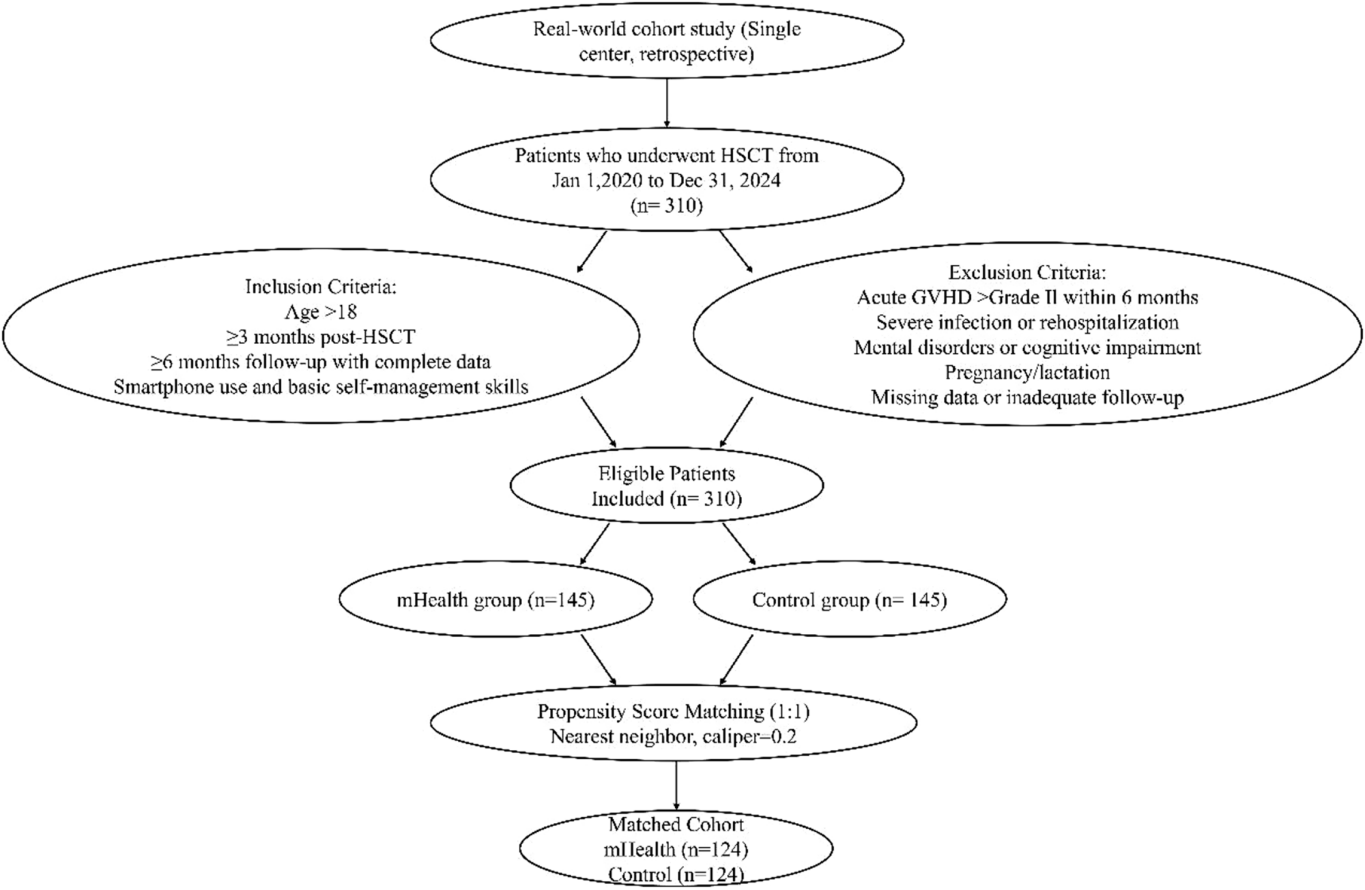
Research design and patient screening process.

**Figure 2:**
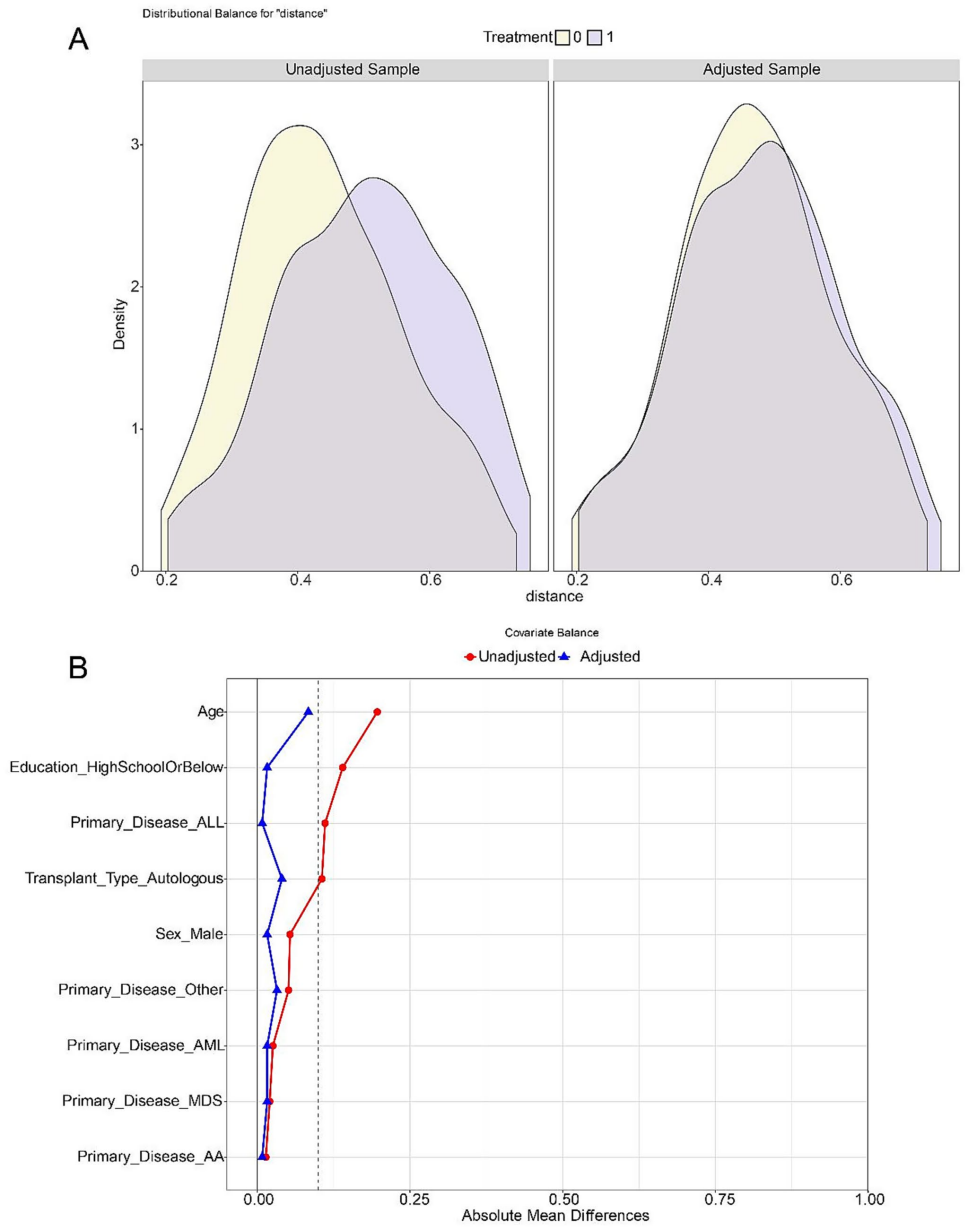
Assessment of baseline balance between the mHealth and control groups before and after propensity score matching (PSM). **(A)** Kernel density plots of the propensity score distribution. After matching (blue), the distribution difference between the mHealth and control groups is markedly reduced compared to the pre-matching state (gray). **(B)** Standardized mean differences (SMD) for baseline covariates. The red dashed line indicates the threshold of SMD = 0.1. After matching, all covariates exhibit SMDs below this threshold, indicating good baseline balance between groups.

To reduce confounding, 1:1 nearest-neighbor propensity score matching (PSM) with a caliper of 0.2 was performed. A total of 310 patients were included before matching (145 in the mHealth group, 165 in the control group). After PSM, 248 patients (124 per group) were retained. Before matching, notable imbalances were observed in age, transplant type, and education level (*p* < 0.1), with standardized mean differences (SMDs) > 0.1. After matching, SMDs for all baseline covariates were below 0.1, indicating satisfactory balance and improved comparability between groups (Table 1 and Figure 1). Figure 2 shows group distributions before and after matching. Kernel density plots of propensity scores indicated improved overlap after matching (Figure 2A). The SMD plot confirmed that all covariates were well balanced (SMD < 0.1), supporting the validity of the matching procedure (Figure 2B).

**Table 1:** Baseline characteristics before and after propensity score matching.

Variable	Before PSM						After PSM					
	Total (*n* = 310)	Control (*n* = 165)	mHealth (*n* = 145)	Statistic	*P*	SMD	Total (*n* = 248)	Control (*n* = 124)	mHealth (*n* = 124)	Statistic	*P*	SMD
Age, Mean ± SD	41.93 ± 11.23	42.93 ± 11.49	40.80 ± 10.85	t = 1.672	0.095	−0.196	41.91 ± 11.37	42.37 ± 11.75	41.46 ± 11.01	t = 0.627	0.531	−0.082
BMI, Mean ± SD	23.70 ± 3.47	23.82 ± 3.55	23.57 ± 3.39	t = 0.643	0.521	−0.075	23.67 ± 3.59	23.95 ± 3.64	23.39 ± 3.53	t = 1.212	0.227	−0.156
Sex, *n* (%)				χ^2^ = 0.911	0.340					χ^2^ = 0.066	0.797	
Female	128 (41.29)	64 (38.79)	64 (44.14)			0.108	106 (42.74)	54 (43.55)	52 (41.94)			−0.033
Male	182 (58.71)	101 (61.21)	81 (55.86)			−0.108	142 (57.26)	70 (56.45)	72 (58.06)			0.033
Transplant type, *n* (%)				χ^2^ = 3.992	0.046					χ^2^ = 0.458	0.498	
Allogeneic	212 (68.39)	121 (73.33)	91 (62.76)			−0.219	167 (67.34)	86 (69.35)	81 (65.32)			−0.085
Autologous	98 (31.61)	44 (26.67)	54 (37.24)			0.219	81 (32.66)	38 (30.65)	43 (34.68)			0.085
Primary disease, *n* (%)				χ^2^ = 5.194	0.268					χ^2^ = 0.629	0.960	
AA	49 (15.81)	25 (15.15)	24 (16.55)			0.038	41 (16.53)	21 (16.94)	20 (16.13)			−0.022
ALL	91 (29.35)	57 (34.55)	34 (23.45)			−0.262	65 (26.21)	32 (25.81)	33 (26.61)			0.018
AML	92 (29.68)	47 (28.48)	45 (31.03)			0.055	72 (29.03)	37 (29.84)	35 (28.23)			−0.036
MDS	33 (10.65)	16 (9.70)	17 (11.72)			0.063	30 (12.1)	16 (12.90)	14 (11.29)			−0.051
Other	45 (14.52)	20 (12.12)	25 (17.24)			0.136	40 (16.13)	18 (14.52)	22 (17.74)			0.084
Education, *n* (%)				χ^2^ = 6.159	0.013					χ^2^ = 0.066	0.797	
College or above	133 (42.9)	60 (36.36)	73 (50.34)			0.280	106 (42.74)	52 (41.94)	54 (43.55)			0.033
High school or below	177 (57.1)	105 (63.64)	72 (49.66)			−0.280	142 (57.26)	72 (58.06)	70 (56.45)			−0.033
Urban residence, *n* (%)				χ^2^ = 0.560	0.454					χ^2^ = 1.348	0.246	
No	81 (26.13)	46 (27.88)	35 (24.14)			−0.087	64 (25.81)	36 (29.03)	28 (22.58)			−0.154
Yes	229 (73.87)	119 (72.12)	110 (75.86)			0.087	184 (74.19)	88 (70.97)	96 (77.42)			0.154
Chronic disease, *n* (%)				χ^2^ = 0.238	0.625					χ^2^ = 0.930	0.335	
No	253 (81.61)	133 (80.61)	120 (82.76)			0.057	200 (80.65)	97 (78.23)	103 (83.06)			0.129
Yes	57 (18.39)	32 (19.39)	25 (17.24)			−0.057	48 (19.35)	27 (21.77)	21 (16.94)			−0.129
Letermovir prophylaxis, *n* (%)				χ^2^ = 0.051	0.821					χ^2^ = 0.170	0.682	
No	217 (70.00)	115 (69.70)	102 (70.34)			0.014	175 (70.56)	86 (69.35)	89 (71.77)			0.052
Yes	93 (30.00)	50 (30.30)	43 (29.66)			−0.014	73 (29.44)	38 (30.65)	35 (28.23)			−0.052

SD, standard deviation; BMI, body mass index, AA, aplastic anemia, ALL, acute lymphoblastic leukemia; AML, acute myeloid leukemia; MDS, myelodysplastic syndrome.

### 3.2 Primary outcome analysis

**Figure 3:**
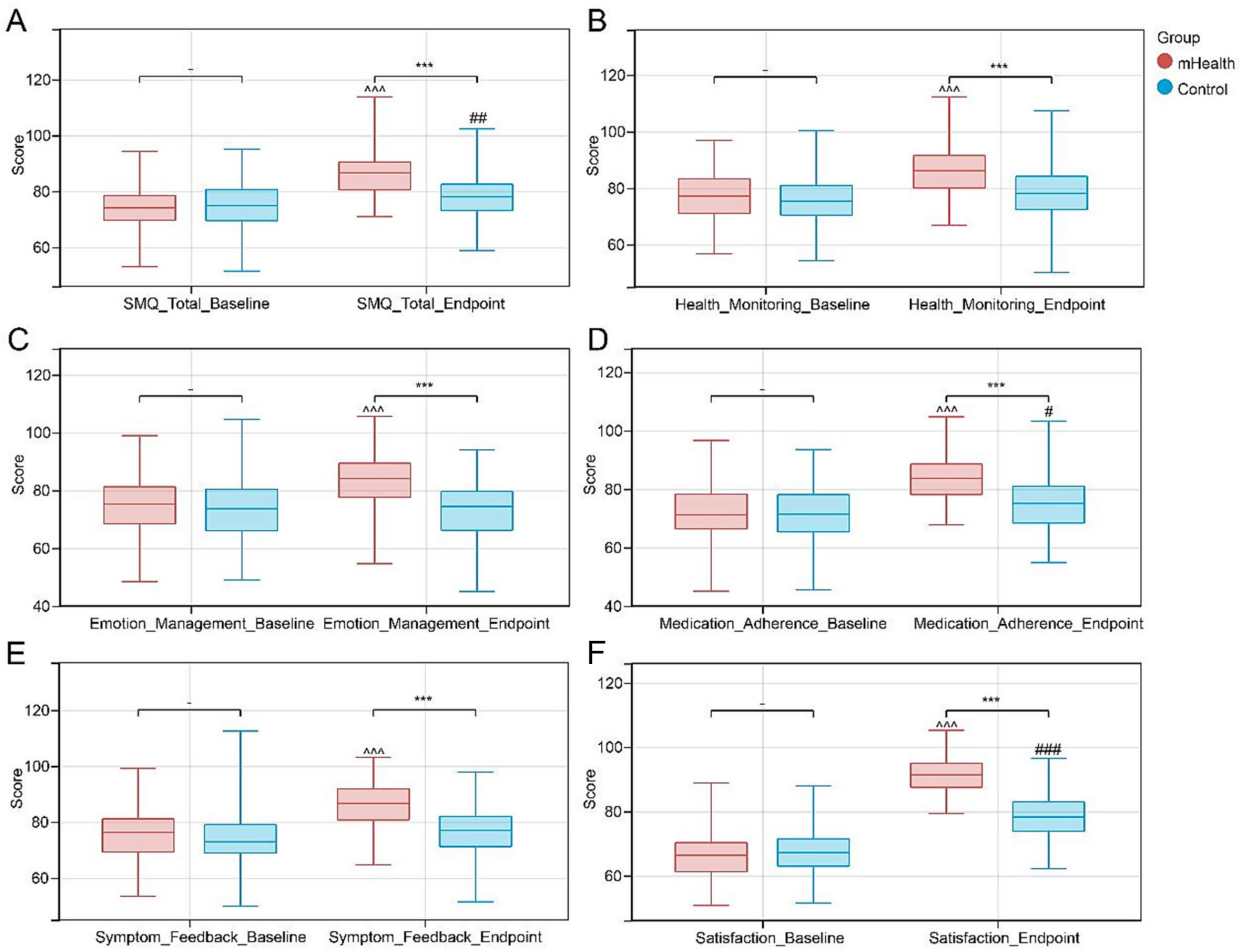
Improvements in symptom management and patient satisfaction. **(A)** Symptom management questionnaire (SMQ) total score; **(B)** health monitoring; **(C)** emotional regulation; **(D)** medication adherence; **(E)** symptom reporting; **(F)** patient satisfaction. ***p < 0.001 between-group comparison. #*p* < 0.05, ##*p* < 0.01; ###*p* < 0.001 within control group pre- vs. post-intervention. ^^^*p* < 0.001 within mHealth group pre- vs. post-intervention.

At the six-month follow-up, patients in the mHealth group appeared to show greater improvements in both quality of life and self-efficacy compared to the control group (Table 2 and Figures 3A–F); the mean EORTC QLQ-C30 score increased from 73.71 ± 10.06 to 83.69 ± 8.45 (*p* < 0.001) in the mHealth group, whereas only a minor increase was observed in the control group (to 74.18 ± 10.56), and similarly, the SME score in the mHealth group improved from 8.43 ± 1.27 to 10.44 ± 1.13 (*p* < 0.001), with a between-group difference of *p* = 0.026, suggesting that the mHealth intervention may be associated with better patient-reported quality of life and self-management efficacy (both *p* < 0.001 for between-group comparison). Complications were analyzed stratified by autologous and allogeneic hematopoietic stem cell transplantation (Table 3); no GVHD occurred in autologous transplant patients, while in the allogeneic transplant subgroup, the mHealth group had a lower observed incidence of GVHD compared to the control group (16.05% vs. 26.74%, χ^2^ = 4.02, *p* = 0.045), and lower infection rates in the mHealth group were observed in both the autologous (χ^2^ = 4.21, *p* = 0.040) and allogeneic (χ^2^ = 6.01, *p* = 0.014) transplant subgroups. A significant inter-group difference in CMV reactivation was only observed in the allogeneic transplant subgroup (χ^2^ = 9.49, *p* = 0.002), while no statistical significance was found in the autologous transplant subgroup (χ^2^ = 1.90, *p* = 0.168), and abnormal liver function and emotional disorders showed a trend toward reduction in all subgroups, though the differences were not statistically significant. Overall comparison showed that compared with the control group, the mHealth group exhibited numerically lower incidences of GVHD (10.48% vs. 18.55%, χ^2^ = 3.25, *p* = 0.071), infection (13.71% vs. 30.65%, χ^2^ = 10.30, *p* = 0.001), and CMV reactivation (4.84% vs. 18.55%, χ^2^ = 11.29, *p* < 0.001); among these, the differences in infection and CMV reactivation were statistically significant, and the incidence of emotional disorders also appeared to decrease (12.90% vs. 22.58%, χ^2^ = 3.98, *p* = 0.046). Although abnormal liver function showed a decreasing trend in the intervention group, the difference was not statistically significant, and these results suggest that the mobile health-based follow-up management model may be associated with favorable changes in quality of life, self-efficacy, and the incidence of some complications.

**Table 2:** Scores of primary endpoints before and after management in both groups.

Outcome	Baseline (mHealth)	6 Months (mHealth)	Baseline (Control)	6 Months (Control)	Statistics	Between-group comparison (*P*-value)
EORTC QLQ-C30	73.71 ± 10.06	83.69 ± 8.45***	71.56 ± 10.33	74.18 ± 10.56	*t* = −7.84	*P* < 0.001
SME	8.43 ± 1.27	10.44 ± 1.13***	8.56 ± 1.29	8.95 ± 1.48*	*t* = −8.90	*P* < 0.001

*P < 0.05; ***p < 0.001 indicate within-group comparisons before and after intervention. EORTC QLQ-C30: European Organisation for Research and Treatment of Cancer Quality of Life Questionnaire Core 30, SME, self-management efficacy.

**Table 3:** Incidence of complications in overall and stratified by transplant type.

Complication	Overall-mHealth (*n*=124)	Overall-Control (*n*=124)	Overall-χ², *P*	Autologous-mHealth (*n*=43)	Autologous-Control (*n*=38)	Autologous-χ², *P*	Allogeneic-mHealth (*n*=81)	Allogeneic-Control (*n*=86)	Allogeneic-χ², *P*
GVHD, *n* (%)	13 (10.48)	23 (18.55)	3.25, 0.071	0 (0.00)	0 (0.00)	–	13 (16.05)	23 (26.74)	4.02, 0.045
Infection, *n* (%)	17 (13.71)	38 (30.65)	10.30, 0.001	4 (9.30)	10 (26.32)	4.21, 0.040	13 (16.05)	28 (32.56)	6.01, 0.014
CMV reactivation, *n* (%)	6 (4.84)	23 (18.55)	11.29, <0.001	1 (2.33)	4 (10.53)	1.90, 0.168	5 (6.17)	19 (22.09)	9.49, 0.002
Liver dysfunction, *n* (%)	9 (7.26)	18 (14.52)	3.37, 0.067	2 (4.65)	5 (13.16)	1.80, 0.180	7 (8.64)	13 (15.12)	1.75, 0.186
Emotional disorders, *n* (%)	16 (12.90)	28 (22.58)	3.98, 0.046	5 (11.63)	8 (21.05)	1.39, 0.238	11 (13.58)	20 (23.26)	2.64, 0.104

GVHD, graft-versus-host disease; CMV, cytomegalovirus.

### 3.3 Secondary outcome analysis

**Figure 4:**
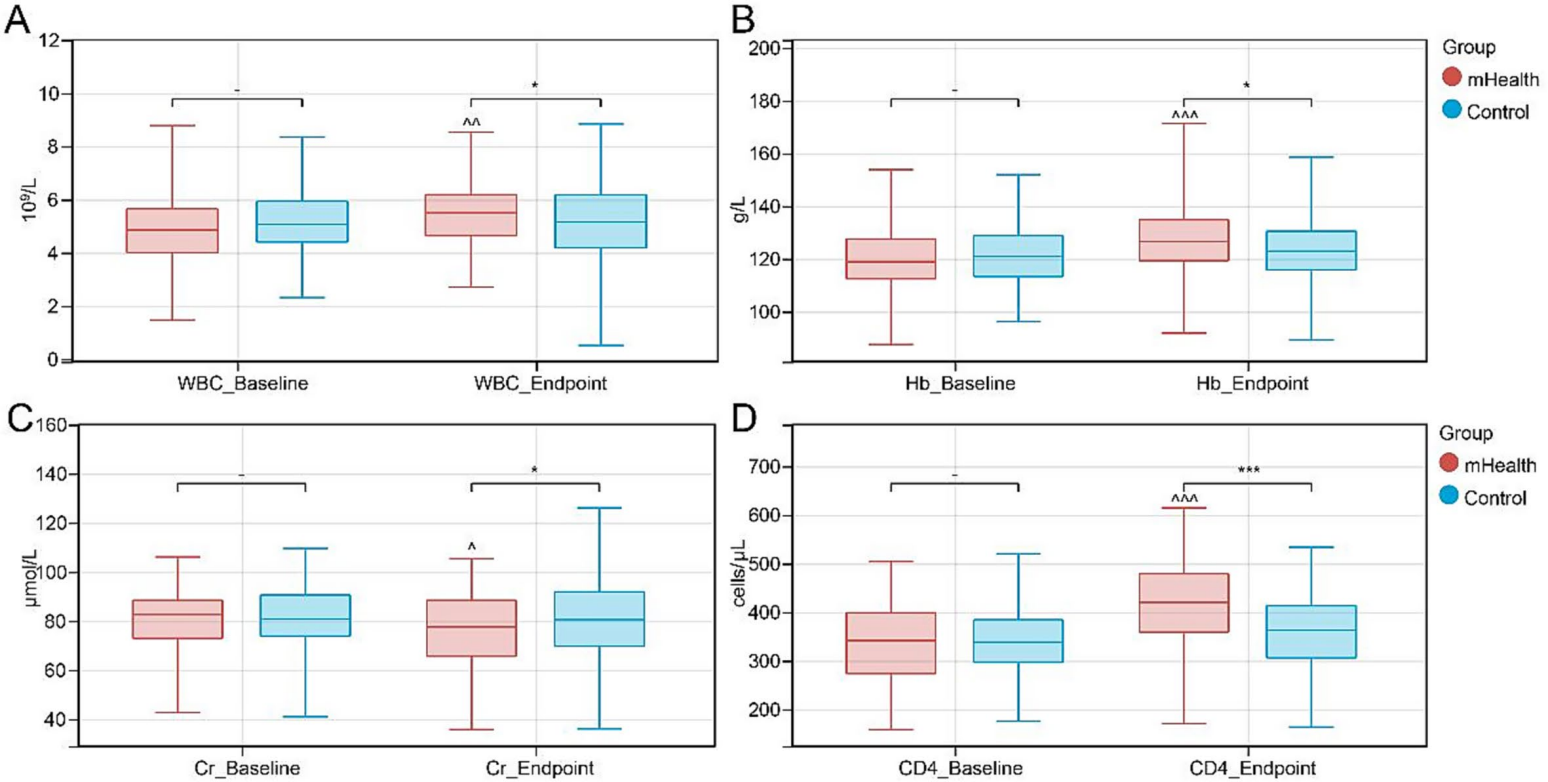
Trends in clinical biomarkers. **(A)** White blood cell (WBC) count; **(B)** Hemoglobin (Hb) level; **(C)** Serum creatinine (Cr) level; **(D)** CD4-positive T lymphocyte (CD4^+^ T-cell) count. ^*^p < 0.05, ***p < 0.001 between-group comparison. ^*p* < 0.05; ^^^*p* < 0.001 within mHealth group pre- vs. post-intervention.

At the 6-month follow-up, the mHealth group demonstrated greater improvement in overall symptom management, with the total SMQ score increasing from 74.03 ± 7.76 to 86.32 ± 7.46 (*p* < 0.001), whereas the control group showed only a modest increase from 75.12 ± 8.55 to 77.96 ± 7.32 (*p* = 0.004), with a statistically significant between-group difference (*p* < 0.001); across all subdomains, the mHealth group exhibited more favorable improvements (health monitoring: 85.75 ± 8.30 vs. 77.39 ± 8.55, medication adherence: 83.81 ± 7.81 vs. 71.77 ± 9.76, emotional regulation: 83.30 ± 9.55 vs. 75.25 ± 9.74, and symptom reporting: 86.51 ± 8.04 vs. 76.04 ± 9.34), all with *p* < 0.001 compared to baseline, while improvements in the control group were limited across these domains. Patient satisfaction increased markedly in the intervention group (from 66.30 ± 6.95 to 91.15 ± 5.34, *p* < 0.001), exceeding that in the control group (from 66.70 ± 6.84 to 78.45 ± 6.67, *p* < 0.001), with a significant between-group difference (*p* < 0.001), and these findings suggest that mHealth interventions may contribute to a more favorable patient experience. In terms of laboratory outcomes, the mHealth group showed more favorable changes, including increases in white blood cell count (WBC, *p* = 0.002), hemoglobin (Hb, *p* < 0.001), and CD4 + T-cell count (CD4, *p* < 0.001), alongside a reduction in serum creatinine (Cr, *p* = 0.018), all of which appeared to be more favorable than changes observed in the control group, and these results imply potential benefits of mHealth interventions in supporting immune function and preserving renal function (Figures 4A–D). Regarding medical resource utilization, the mHealth group appeared to have more favorable efficiency compared to the control group, with lower observed readmission rates and fewer daily nursing unit visits (Table 4); overall, the readmission rate was 8.06% (10/124) in the mHealth group, which was lower than 18.55% (23/124) in the control group (χ^2^ = 6.78, *p* = 0.009), and the average daily number of nursing unit visits was (0.12 ± 0.05) in the mHealth group, fewer than (0.25 ± 0.08) in the control group (t = 13.52, *p* < 0.001).

**Table 4:** Comparison of medical resource utilization between the two groups.

Indicators of medical resource utilization	mHealth group	Control group	Statistic	*P* value
Readmission rate, *n* (%)	10 (8.06)	23 (18.55)	χ^2^ = 6.78	*P* = 0.009
Daily nursing visits, Mean±SD	0.12 ± 0.05	0.25 ± 0.08	*t* = 13.52	*P* < 0.001

### 3.4 Multivariate linear regression analysis

**Figure 5:**
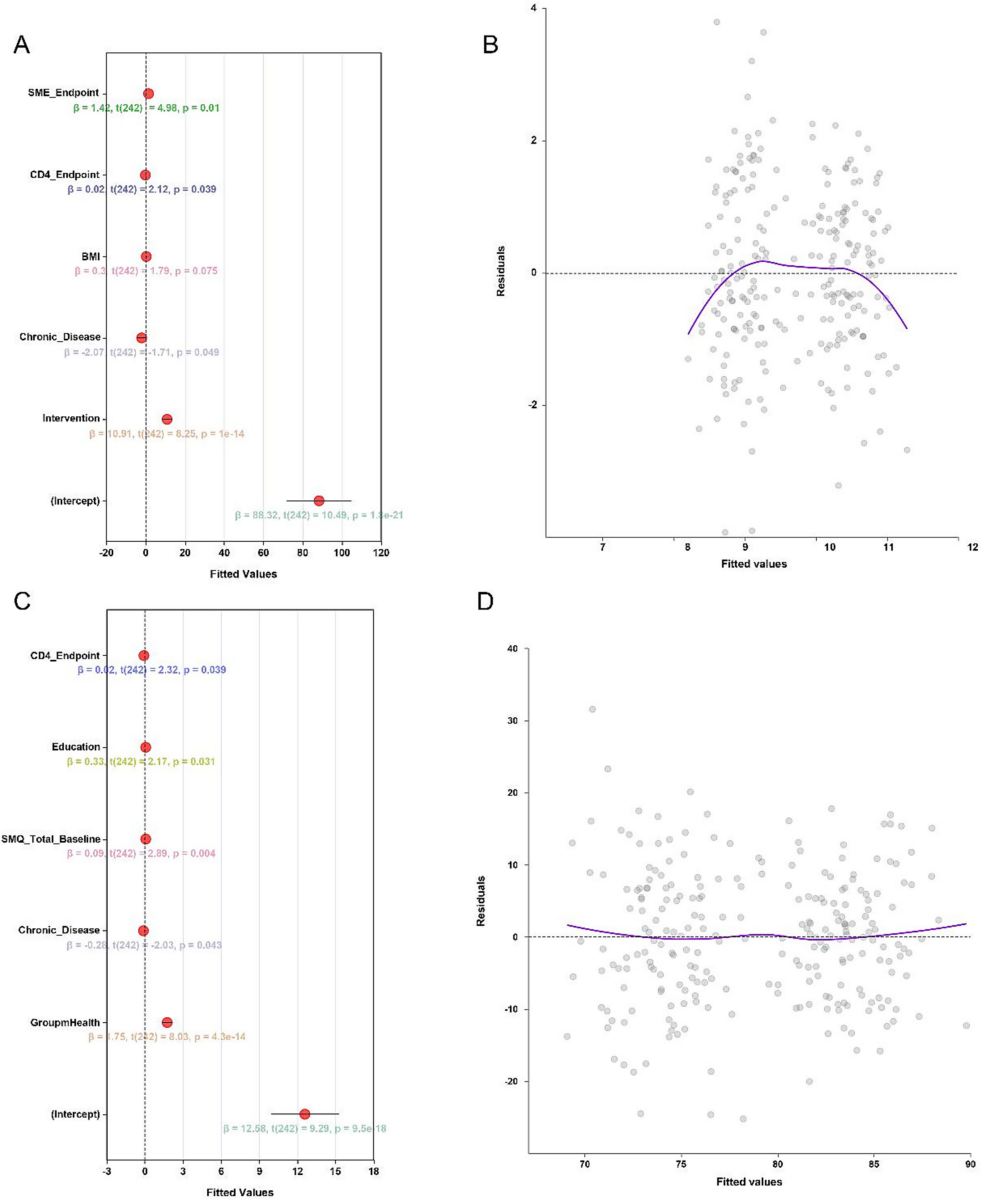
Multivariate linear regression model results: **(A)** Regression coefficients for predictors of European Organization for Research and Treatment of Cancer Quality of Life Questionnaire Core 30 (EORTC QLQ-C30); **(B)** Residual distribution for the EORTC model; **(C)** Regression coefficients for self-management efficacy predictors (SME model); **(D)** Residual distribution for the SME model.

With higher quality of life (*β* = 10.91, *p* < 0.001) and self-management efficacy (SME; *β* = 1.75, *p* < 0.001). In the quality-of-life model, terminal CD4 + count (*β* = 0.02, *p* = 0.039) and SME score (*β* = 1.42, *p* = 0.010) were positively associated with favorable outcomes, whereas the presence of chronic comorbidities showed a negative association (*β* = −2.07, *p* = 0.049). In the SME model, educational attainment (*β* = 0.33, *p* = 0.031), baseline SMQ score (*β* = 0.09, *p* = 0.004), and terminal CD4 + count (*β* = 0.02, *p* = 0.039) were identified as potential independent predictors, while chronic disease remained inversely associated (*β* = −0.28, *p* = 0.043). Model diagnostics supported adequate model fit, with approximately evenly distributed residuals (Figures 5A–D).

### 3.5 Univariate and multivariate logistic regression analysis

**Figure 6:**
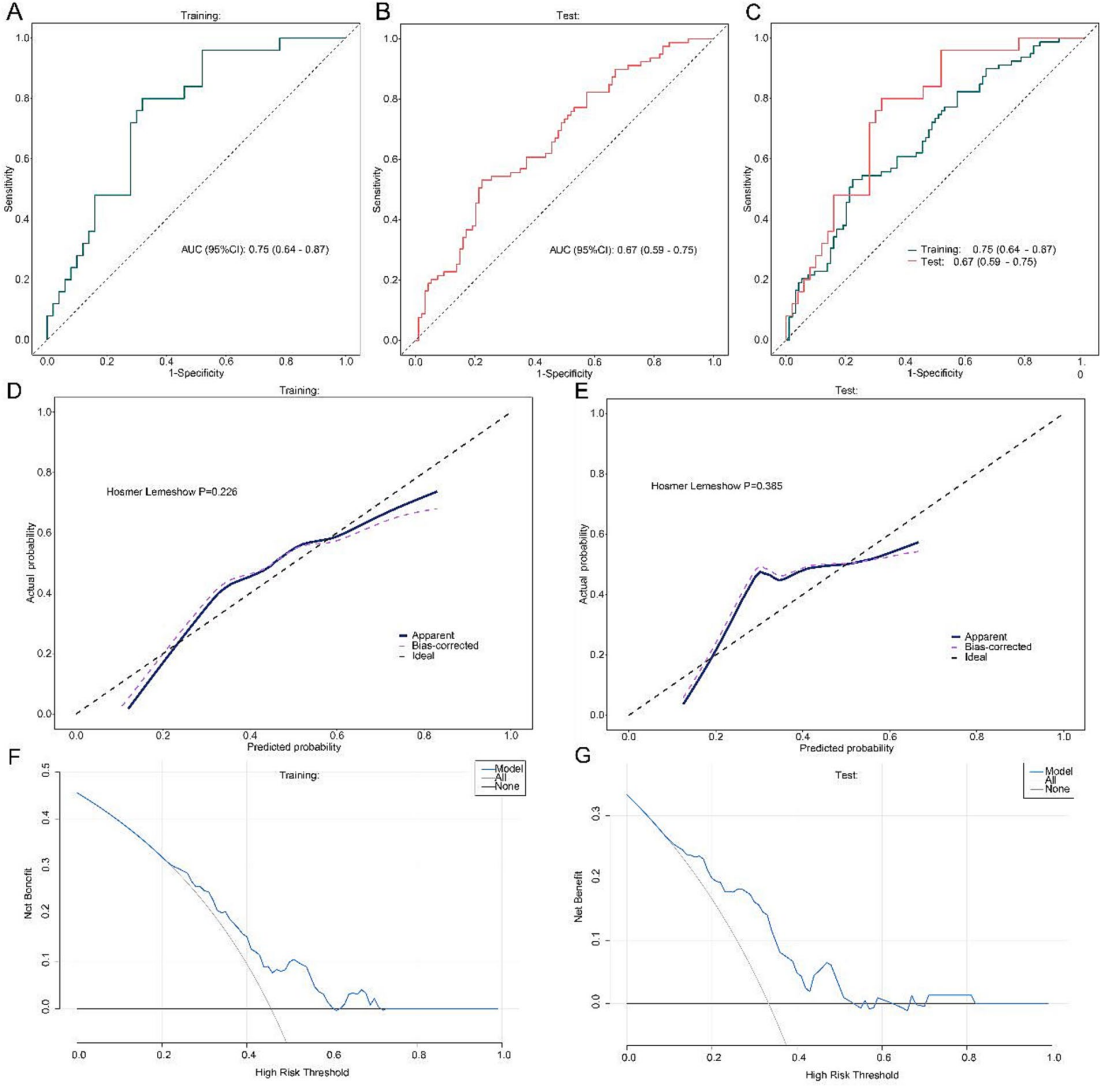
Evaluation of logistic regression model performance. **(A)** Receiver operating characteristic (ROC) curve (training set); **(B)** ROC curve (test set); **(C)** Combined ROC comparison; **(D)** Calibration curve (training set); **(E)** Calibration curve (test set); **(F)** Decision curve analysis (training set); **(G)** Decision curve analysis (test set).

To explore the independent association between mHealth intervention and complication risk, logistic regression models were constructed. Univariate analysis indicated that higher body mass index (BMI) (OR = 0.89; 95% CI: 0.81–0.97; *p* = 0.010), baseline CD4 + count (per 10/μL increase, OR = 0.96; *p* = 0.045), total symptom management score (SMQ) (OR = 0.95; *p* = 0.007), health monitoring ability (OR = 0.95; *p* = 0.017), and emotional regulation (OR = 0.96; *p* = 0.028) may be related to lower odds of complications. In addition, mHealth intervention showed a potential protective association (OR = 0.40; *p* = 0.004). In the multivariate model, mHealth intervention remained independently associated with lower complication risk (OR = 0.40; 95% CI: 0.19–0.83; *p* = 0.011), along with BMI (OR = 0.87; *p* = 0.005), endpoint CD4 + count (OR = 0.97; *p* = 0.042), and health monitoring capacity (OR = 0.96; *p* = 0.036), while total SMQ score and emotional regulation showed borderline associations (*p* = 0.059 and 0.051, respectively). The model demonstrated acceptable predictive performance, with an AUC of 0.75 (95% CI: 0.64–0.87) in the training set and 0.67 (95% CI: 0.59–0.75) in the test set; the Hosmer–Lemeshow test suggested acceptable calibration (*p* = 0.226 for training; *p* = 0.385 for test), and decision curve analysis (DCA) indicated potential clinical utility and net benefit across a range of threshold probabilities (Figures 6A–G).

### 3.6 Subgroup analysis

**Figure 7:**
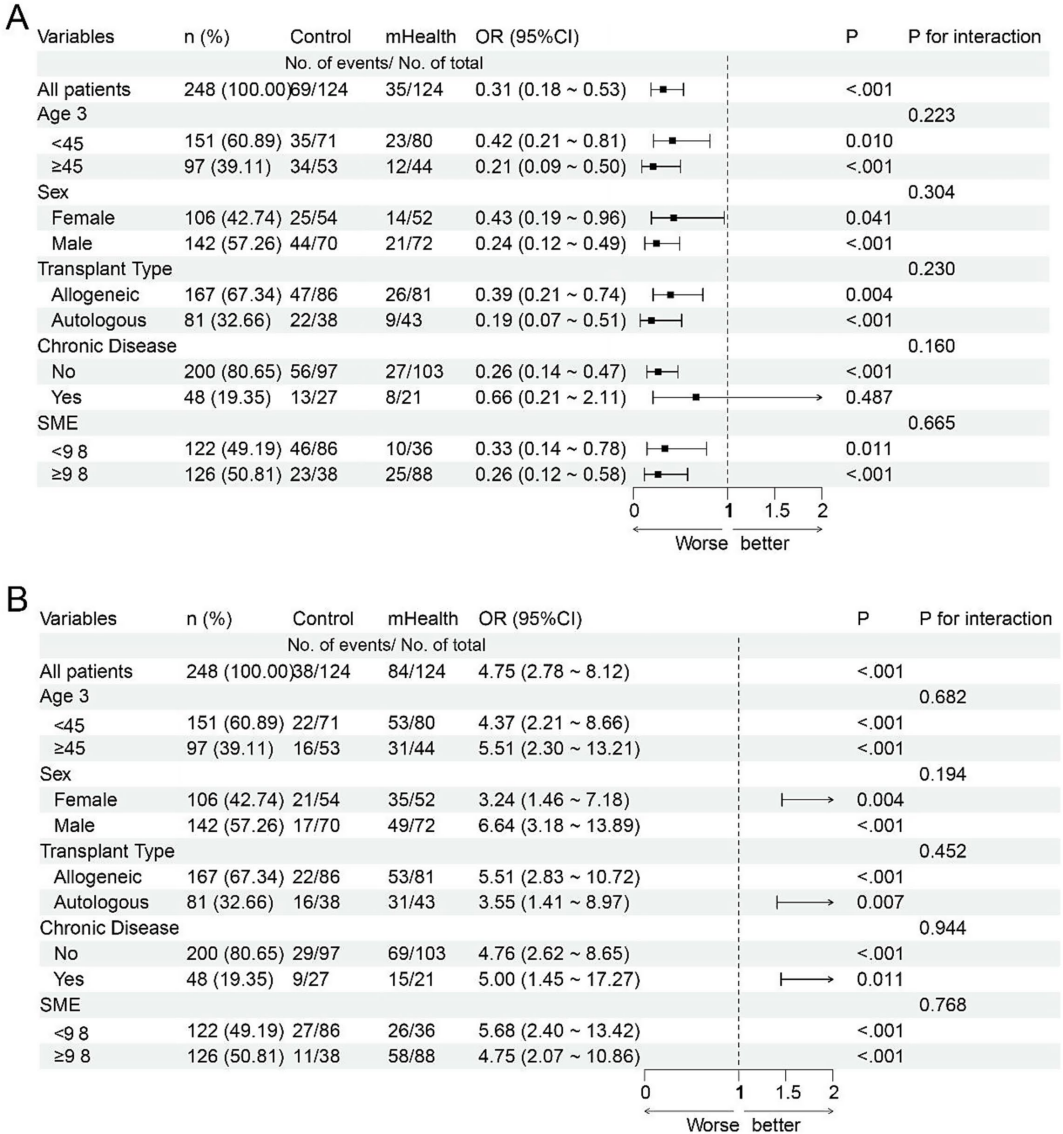
Forest plots of subgroup analyses. **(A)** Subgroup effects of mHealth intervention on quality-of-life improvement. **(B)** Subgroup effects of mHealth intervention on complication risk reduction.

To evaluate the consistency of associations related to mHealth intervention across patient subgroups, subgroup analyses were performed for quality-of-life improvement and complication incidence (Figures 7A,B). In the overall sample, mHealth was associated with a greater likelihood of quality-of-life improvement (OR = 4.75; 95% CI: 2.78–8.12; *p* < 0.001) and lower complication risk (OR = 0.31; 95% CI: 0.18–0.53; *p* < 0.001). Stratified analyses indicated that the observed associations between mHealth and better quality of life were consistent across subgroups defined by age, sex, transplant type, chronic conditions, and baseline SME level (all ORs > 3.2; *p* < 0.01), with no significant interaction terms (all *p* > 0.05), suggesting no substantial effect modification by these characteristics. Similarly, mHealth intervention was associated with lower complication risk across all subgroups (all ORs < 0.5; *p* < 0.05), again without significant interactions. These findings may support the potential generalizability and consistency of mHealth-related associations across diverse patient populations.

### 3.7 Analysis of mHealth application participation

During the intervention period, all 124 patients in the mHealth group completed initial registration and application training, yielding a 100% retention rate, although variability in usage patterns was observed (Table 5). The mean daily application usage duration was 12.35 ± 4.28 min, with a mean weekly frequency of 5.23 ± 1.67 sessions; usage was highest during the first 1–3 months after transplantation (6.12 ± 1.35 sessions per week) and slightly declined during 3–6 months (4.38 ± 1.52 sessions per week) while remaining relatively high (≥4 sessions per week). Regarding functional usage preferences, the most frequently used functions were health monitoring (92.74%, 115/124), symptom feedback (88.71%, 109/124), and medication reminders (85.48%, 106/124), whereas lower usage was observed for emotion diary (62.10%, 77/124) and online consultation (58.87%, 73/124). A total of 18 patients (14.52%) exhibited some degree of technical fatigue, characterized by reduced usage frequency and engagement, all occurring after 3 months post-transplant; all patients resumed regular use following targeted remote support, with no discontinuations due to technical fatigue. Low participation was defined as weekly usage < 3 times and daily duration < 5 min, identifying 28 patients (22.58%) in the low-participation subgroup; these patients were more likely to be aged ≥50 years (71.43%, 20/28), have education at or below senior high school (82.14%, 23/28), and receive allogeneic transplantation (75.00%, 21/28), with significant differences compared with the high-participation subgroup (all *p* < 0.05), whereas gender, BMI, and comorbidities showed no significant associations with participation level (all *p* > 0.05).

**Table 5:** Participation of patients in the mHealth group in the application.

Participation indicators	Total (*n* = 124)	Autologous transplant (*n* = 43)	Allogeneic transplant (*n* = 81)	Statistic, *P* value
Average daily usage duration (minutes), Mean±SD	12.35 ± 4.28	13.82 ± 4.51	11.56 ± 4.03	t = 2.31, *p* = 0.023
Average weekly usage frequency (times), Mean±SD	5.23 ± 1.67	5.87 ± 1.52	4.89 ± 1.65	t = 2.85, *P* = 0.005
Function usage rate, *n* (%)				
Health monitoring	115 (92.74)	40 (93.02)	75 (92.59)	χ^2^ = 0.01, *p* = 0.921
Symptom feedback	109 (88.71)	39 (90.70)	70 (86.42)	χ^2^ = 0.45, *p* = 0.502
Medication reminders	106 (85.48)	37 (86.05)	69 (85.19)	χ^2^ = 0.02, *p* = 0.887
Emotion diary	77 (62.10)	30 (69.77)	47 (58.02)	χ^2^ = 2.13, *p* = 0.144
Online consultation	73 (58.87)	28 (65.12)	45 (55.56)	χ^2^ = 1.12, *p* = 0.290
Incidence of technical fatigue, *n* (%)	18 (14.52)	3 (6.98)	15(18.52)	χ^2^ = 3.97, *P* = 0.046
Low-participation patients, *n* (%)	28 (22.58)	7 (16.28)	21(25.93)	χ^2^ = 1.52, *p* = 0.218
Characteristics of low-participation subgroup, *n* (%)				
Age ≥50 years	20 (71.43)	5 (71.43)	15 (71.43)	χ^2^ = 0.00, *p* = 1.000
Education level: senior high school or below	23 (82.14)	6 (85.71)	17 (80.95)	χ^2^ = 0.12, *p* = 0.728

## 4 Discussion

The results of this study indicate that mobile health (mHealth)-based follow-up management is associated with improvements in quality of life, self-efficacy, and symptom management, as well as a reduction in the incidence of complications such as infections, cytomegalovirus (CMV) reactivation, and emotional disorders among patients undergoing hematopoietic stem cell transplantation (HSCT). Importantly, a core aspect of interpreting the findings of this study is focusing on the independent effects of individual mHealth components, which should also be a priority for future research. The mHealth platform used in this study integrated four core components: health data monitoring, intelligent medication management, personalized health education, and psychological support. However, the independent contributions of each component to patient health outcomes have not yet been analyzed. Clarifying the differences in the effects of different components can not only accurately interpret the core mechanism underlying the “superiority of mHealth intervention over routine follow-up” observed in this study but also provide a basis for optimizing platform design and focusing on key intervention modules in future studies, which is consistent with the research consensus in the eHealth field of “precisely decomposing digital intervention components to improve intervention efficiency” (30).

Previous studies have noted that although HSCT can effectively treat hematological diseases, patients often experience reduced quality of life, long-term treatment sequelae, and increased psychological burden after transplantation (31, 32). Traditional offline follow-up models have limitations such as delayed monitoring, fragmented health guidance, and insufficient patient adherence, making it difficult to meet the long-term rehabilitation management needs of patients (22). In recent years, mHealth interventions have been increasingly applied in the management of HSCT patients, serving as an important supplement to traditional follow-up. However, most existing studies are small-sample pilot/feasibility studies that focus primarily on evaluating overall effects, with insufficient decomposition and mechanism verification of specific components (e.g., dynamic complication monitoring, immune function prompts, and individualized medication reminders) (19, 22).

In a study by Racioppi et al., a dedicated mHealth application (TRU-BMT) was developed specifically for transplant patients to facilitate daily symptom tracking and health monitoring, aiming to detect adverse events early and enable timely intervention (19). High adherence to the app was associated with shorter hospital stays and favorable outcomes such as a lower incidence of chronic graft-versus-host disease (GVHD), supporting the feasibility and clinical value of mHealth in HSCT care (19). Consistent with these findings, our study showed that patients in the mHealth group had significantly higher EORTC QLQ-C30 quality of life scores and significantly improved self-efficacy after six months of intervention (both *p* < 0.001), outperforming the control group.

These findings align with evidence from other clinical contexts. For example, a recent meta-analysis in postoperative cancer patients found that smartphone-based interventions significantly improved quality of life [standardized mean difference (SMD) = −0.58] and enhanced patient self-efficacy (SMD = 0.90) (33). Similarly, mHealth apps have been shown to significantly enhance self-efficacy in patients following cardiac surgery (34). Collectively, these studies suggest that mHealth interventions can improve patient self-management through continuous health education, real-time feedback, and structured care guidance, thereby improving both health-related quality of life and confidence in disease management. The substantial benefits observed in our study may be attributable to the integration of health monitoring, medication reminders, and psychological support throughout the intervention period.

With regard to complications, the mHealth group exhibited significantly lower rates of infection (13.71% vs. 30.65%), CMV reactivation (4.84% vs. 18.55%), and emotional disorders (12.90% vs. 22.58%) compared to controls. While trends toward reduced GVHD and liver function impairment were observed, these did not reach statistical significance. These results indicate that mHealth interventions may optimize self-management and reduce preventable complications. Literature has emphasized that strict adherence to immunosuppressive regimens and infection prevention strategies post-transplantation is critical for reducing infectious complications (35, 36). For instance, a study focusing on post-transplant medication adherence in pediatric patients reported that non-adherence was associated with a higher rate of infections and adverse outcomes (35). mHealth apps, functioning as “virtual assistants,” can support patients and caregivers by providing medication reminders and facilitating accurate health record-keeping. Our platform incorporated medication management and follow-up communication features, which may have improved adherence and early symptom recognition, contributing to the observed reduction in infections and CMV reactivation. Moreover, improvements in laboratory parameters such as elevated WBC, Hb, and CD4 + counts, alongside a reduction in serum creatinine, suggest a potential role of mHealth in supporting immune recovery and preserving renal function. Studies on immune reconstitution have shown that early recovery of lymphocytes—particularly CD4 + T cells—is closely associated with favorable HSCT outcomes (37). The higher CD4 + levels observed in the mHealth group in our study may indicate more robust immune reconstitution, which corresponds with the lower complication rates and improved risk indicators for long-term survival (although survival outcomes were not measured due to the six-month follow-up window). Finally, the significantly reduced incidence of emotional disorders in the mHealth group may reflect the benefit of sustained health education and integrated psychological support. Previous research has shown that mHealth interventions can alleviate anxiety and depressive symptoms in patients (33), consistent with our findings of reduced emotional distress.

Patients in the mHealth group demonstrated significantly greater improvements in overall symptom management scores (SMQ) and all subdomains—including health monitoring, medication adherence, emotional regulation, and symptom reporting—compared with the control group. These findings suggest that a structured mobile follow-up system can effectively facilitate patients’ real-time health monitoring and timely communication of clinical issues. Similarly, a study by Li et al. found that smartphone-based follow-up platforms improved symptom management and rehabilitation guidance among postoperative patients, contributing to better functional outcomes and overall health status (38). In terms of patient satisfaction, scores were significantly higher in the mHealth group than in the control group, consistent with prior reports highlighting the high acceptability of mHealth interventions. For example, Lee et al. reported a mean satisfaction score of 4.22 out of 5 among breast cancer survivors participating in an mHealth-based exercise intervention (39). In line with these findings, our data indicate that a personalized, responsive mHealth platform markedly enhances patients’ healthcare experience and satisfaction, likely by fulfilling their needs for continuous care and psychosocial support.

Multivariate regression analyses further confirmed the independent effect of the mHealth intervention. Even after adjusting for potential confounders such as age, education level, and chronic disease history, the intervention remained a significant predictor of improved quality of life (*β* = 10.91, *p* < 0.001) and self-management efficacy (*β* = 1.75, *p* < 0.001). Additionally, higher CD4 + T-cell levels and baseline self-efficacy scores were positively associated with better post-intervention outcomes, while the presence of chronic comorbidities was negatively associated with both endpoints. These findings align with previous research indicating that individuals with chronic conditions often experience a higher health burden and lower quality of life (37, 40). Subgroup analyses demonstrated the consistent benefits of mHealth across diverse patient populations. Significant improvements in quality of life (all ORs > 3.2, *p* < 0.01) and reductions in complication risk (all ORs < 0.5, *p* < 0.05) were observed across subgroups defined by age, sex, transplant type, chronic disease status, and baseline self-efficacy level. No significant interaction effects were detected, suggesting that the effectiveness of the mHealth model is broadly applicable and not dependent on baseline characteristics or transplantation modality.

This study explored the impact of an mHealth-based follow-up management model on the health outcomes of HSCT patients. Although the mHealth group showed advantages over the control group in terms of quality of life, self-efficacy, complication prevention and control, and medical resource utilization, several limitations should be acknowledged. The primary methodological limitation is the self-selection bias inherently linked to voluntary participation in the mHealth intervention. Patients in the mHealth group were voluntarily recruited, and required to actively register on the platform, possess basic smartphone operation skills, and complete ≥6 months of continuous follow-up. These enrollment criteria meant participants likely had intrinsically higher health management motivation, better digital literacy, and stronger willingness for self-management than the standard-care control group. Critically, these factors can independently affect core study outcomes—including quality of life, self-efficacy, and complication incidence—regardless of the mHealth intervention itself. The exclusion of 8 patients due to insufficient APP operation ability further confirms the presence of this selection bias, indicating the observed benefits may be partially attributed to patients’ inherent characteristics rather than the intervention effect alone. Although propensity score matching (PSM) balanced the baseline demographic and clinical characteristics of the two groups (SMD of all included covariates < 0.1 after matching, Table 1), baseline metrics closely associated with self-selection—including quality of life (EORTC QLQ-C30), self-efficacy (SME), and treatment adherence—were not included in the matching model. This may have amplified residual bias from inherent between-group differences, limiting the reliability of causal inference, consistent with limitations reported in related mHealth studies. Additionally, the reasons for non-registration on the mHealth platform among control patients were not systematically analyzed, which may affect the accuracy of result interpretation. Furthermore, the retrospective matched design lacks high-level randomized evidence; only stable post-transplant patients were included, with a 6-month follow-up period, which precludes evaluation of early post-transplant and long-term efficacy, and leaves potential unmeasured confounding unresolved.

To address the above limitations, targeted improvements are needed in future studies: conducting prospective randomized controlled trials (RCTs) to fundamentally mitigate self-selection bias and clarify the causal effect of the mHealth intervention; expanding the sample size and conducting multi-center studies to enhance generalizability; extending the follow-up period to 12–24 months to verify long-term efficacy; enrolling patients from the day of stem cell infusion to cover the high-incidence period of post-transplant complications; systematically collecting baseline data on quality of life, self-efficacy, and adherence, and incorporating these into PSM models to reduce residual confounding; exploring the independent roles of different mHealth functional modules and optimizing the platform to improve engagement among low-participation subgroups; and focusing on clinical promotion feasibility and adherence barriers to design patient-centered applications. The conclusions of this study are only applicable to explaining the association between mHealth intervention and improved quality of life, self-management ability, and reduced healthcare utilization in HSCT patients, and cannot be directly extrapolated to infer a causal preventive effect on organ-specific complications.

## 5 Conclusion

The results of this study suggest that the mHealth-based follow-up management model is associated with improved quality of life, self-management efficacy, and patient satisfaction, as well as a lower incidence of common post-transplant complications including infections and CMV reactivation among HSCT patients. Multivariable regression and subgroup analyses further indicate that these observed associations are independent of measured baseline confounders, and remained consistent among patients of different ages, genders, and transplant types. It is important to emphasize that the most critical limitation of this study is the self-selection bias inherent to voluntary recruitment of the mHealth group. Patients enrolled in the mHealth program typically had higher health management motivation, greater engagement, better digital literacy, and potentially more favorable baseline clinical characteristics than those in the standard-care control group. Therefore, a causal relationship between mHealth intervention and the aforementioned health outcomes cannot be established, and the observed between-group differences may be partially attributed to patients’ inherent characteristics rather than the intervention itself.

As a digital management tool integrating health education, behavioral guidance, and dynamic monitoring, mHealth intervention may represent a potential auxiliary approach for long-term post-transplant health management in HSCT patients, with preliminary observational support. Future studies should conduct multi-center, long-term prospective randomized controlled trials (RCTs) to effectively mitigate selection bias, further validate the sustained associations with long-term prognosis and quality of life, and clarify the causal effect of mHealth intervention. Meanwhile, optimizing platform design to improve engagement and usability among low-participation subgroups, and exploring its integration with smart wearables and artificial intelligence technology, may offer a more robust framework for precise care and individualized intervention in HSCT patients.

## Data Availability

The raw data supporting the conclusions of this article will be made available by the authors, without undue reservation.
