# Supplementary material for: Development and effectiveness evaluation of a mobile health-based follow-up management model for patients after hematopoietic stem cell transplantation
Source: Front Med (Lausanne). 2026 Mar 19;13:1786747. doi: 10.3389/fmed.2026.1786747 (PMC13043397; doi:10.3389/fmed.2026.1786747)
Supplement: Supplementary file 1 [file Supplementary_file_1.docx]

Supplementary Table 1. Baseline Characteristics Before and After Propensity Score Matching

| Variable | Before PSM | | | | | |  | After PSM | | | | | |
| --- | --- | --- | --- | --- | --- | --- | --- | --- | --- | --- | --- | --- | --- |
|  | Total (n = 310) | Control (n = 165) | mHealth (n = 145) | Statistic | *P* | SMD |  | Total (n = 248) | Control (n = 124) | mHealth (n = 124) | Statistic | *P* | SMD |
| Age, Mean ± SD | 41.93 ± 11.23 | 42.93 ± 11.49 | 40.80 ± 10.85 | t=1.672 | 0.095 | -0.196 |  | 41.91 ± 11.37 | 42.37 ± 11.75 | 41.46 ± 11.01 | t=0.627 | 0.531 | -0.082 |
| BMI, Mean ± SD | 23.70 ± 3.47 | 23.82 ± 3.55 | 23.57 ± 3.39 | t=0.643 | 0.521 | -0.075 |  | 23.67 ± 3.59 | 23.95 ± 3.64 | 23.39 ± 3.53 | t=1.212 | 0.227 | -0.156 |
| Sex, n (%) |  |  |  | χ²=0.911 | 0.340 |  |  |  |  |  | χ²=0.066 | 0.797 |  |
| Female | 128 (41.29) | 64 (38.79) | 64 (44.14) |  |  | 0.108 |  | 106 (42.74) | 54 (43.55) | 52 (41.94) |  |  | -0.033 |
| Male | 182 (58.71) | 101 (61.21) | 81 (55.86) |  |  | -0.108 |  | 142 (57.26) | 70 (56.45) | 72 (58.06) |  |  | 0.033 |
| Transplant Type, n (%) |  |  |  | χ²=3.992 | 0.046 |  |  |  |  |  | χ²=0.458 | 0.498 |  |
| Allogeneic | 212 (68.39) | 121 (73.33) | 91 (62.76) |  |  | -0.219 |  | 167 (67.34) | 86 (69.35) | 81 (65.32) |  |  | -0.085 |
| Autologous | 98 (31.61) | 44 (26.67) | 54 (37.24) |  |  | 0.219 |  | 81 (32.66) | 38 (30.65) | 43 (34.68) |  |  | 0.085 |
| Primary Disease, n (%) |  |  |  | χ²=5.194 | 0.268 |  |  |  |  |  | χ²=0.629 | 0.960 |  |
| AA | 49 (15.81) | 25 (15.15) | 24 (16.55) |  |  | 0.038 |  | 41 (16.53) | 21 (16.94) | 20 (16.13) |  |  | -0.022 |
| ALL | 91 (29.35) | 57 (34.55) | 34 (23.45) |  |  | -0.262 |  | 65 (26.21) | 32 (25.81) | 33 (26.61) |  |  | 0.018 |
| AML | 92 (29.68) | 47 (28.48) | 45 (31.03) |  |  | 0.055 |  | 72 (29.03) | 37 (29.84) | 35 (28.23) |  |  | -0.036 |
| MDS | 33 (10.65) | 16 (9.70) | 17 (11.72) |  |  | 0.063 |  | 30 (12.1) | 16 (12.90) | 14 (11.29) |  |  | -0.051 |
| Other | 45 (14.52) | 20 (12.12) | 25 (17.24) |  |  | 0.136 |  | 40 (16.13) | 18 (14.52) | 22 (17.74) |  |  | 0.084 |
| Education, n (%) |  |  |  | χ²=6.159 | 0.013 |  |  |  |  |  | χ²=0.066 | 0.797 |  |
| CollegeOrAbove | 133 (42.9) | 60 (36.36) | 73 (50.34) |  |  | 0.280 |  | 106 (42.74) | 52 (41.94) | 54 (43.55) |  |  | 0.033 |
| HighSchoolOrBelow | 177 (57.1) | 105 (63.64) | 72 (49.66) |  |  | -0.280 |  | 142 (57.26) | 72 (58.06) | 70 (56.45) |  |  | -0.033 |
| Urban Residence, n (%) |  |  |  | χ²=0.560 | 0.454 |  |  |  |  |  | χ²=1.348 | 0.246 |  |
| No | 81 (26.13) | 46 (27.88) | 35 (24.14) |  |  | -0.087 |  | 64 (25.81) | 36 (29.03) | 28 (22.58) |  |  | -0.154 |
| Yes | 229 (73.87) | 119 (72.12) | 110 (75.86) |  |  | 0.087 |  | 184 (74.19) | 88 (70.97) | 96 (77.42) |  |  | 0.154 |
| Chronic Disease, n (%) |  |  |  | χ²=0.238 | 0.625 |  |  |  |  |  | χ²=0.930 | 0.335 |  |
| No | 253 (81.61) | 133 (80.61) | 120 (82.76) |  |  | 0.057 |  | 200 (80.65) | 97 (78.23) | 103 (83.06) |  |  | 0.129 |
| Yes | 57 (18.39) | 32 (19.39) | 25 (17.24) |  |  | -0.057 |  | 48 (19.35) | 27 (21.77) | 21 (16.94) |  |  | -0.129 |

SD: Standard Deviation, BMI: Body Mass Index, AA: Aplastic Anemia, ALL: Acute Lymphoblastic Leukemia, AML: Acute Myeloid Leukemia, MDS: Myelodysplastic Syndrome.

Supplementary Table 2. Scores of Primary Endpoints Before and After Management in Both Groups

| Outcome | Baseline (mHealth) | 6 Months (mHealth) | Baseline (Control) | 6 Months (Control) | Statistics | Between-Group Comparison (P-value) |
| --- | --- | --- | --- | --- | --- | --- |
| EORTC QLQ-C30 | 73.71 ± 10.06 | 83.69 ± 8.45*** | 71.56 ± 10.33 | 74.18 ± 10.56 | t = –7.84 | P < 0.001 |
| SME | 8.43 ± 1.27 | 10.44 ± 1.13*** | 8.56 ± 1.29 | 8.95 ± 1.48* | t = –8.90 | P < 0.001 |

Note: *P < 0.05, ***P < 0.001 indicate within-group comparisons before and after intervention. EORTC QLQ-C30: European Organisation for Research and Treatment of Cancer Quality of Life Questionnaire Core 30, SME: Self-Management Efficacy.

Supplementary Table 3. Incidence of Complications Between Groups

| Complication | mHealth Group (n=124) | Control Group (n=124) | Statistics | P-value |
| --- | --- | --- | --- | --- |
| GVHD, n (%) | 23 (18.55) | 13 (10.48) | χ² = 3.25 | 0.071 |
| Infection, n (%) | 38 (30.65) | 17 (13.71) | χ² = 10.30 | 0.001 |
| CMV Reactivation, n (%) | 23 (18.55) | 6 (4.84) | χ² = 11.29 | <0.001 |
| Liver Dysfunction, n (%) | 18 (14.52) | 9 (7.26) | χ² = 3.37 | 0.067 |
| Emotional Disorders, n (%) | 28 (22.58) | 16 (12.90) | χ² = 3.98 | 0.046 |

GVHD: Graft-Versus-Host Disease, CMV: Cytomegalovirus.
